# Contextual factors influencing medicines-related interventions to support safe transitions for care home residents post hospital discharge: a systematic review and meta-ethnographic synthesis

**DOI:** 10.1007/s11096-022-01507-3

**Published:** 2022-11-17

**Authors:** Janani Kandiah, Hamde Nazar, Jeanette Blacklock, Anna Robinson, David Wright

**Affiliations:** 1 University of East Anglia, Norwich Research Park, Norwich, UK; 2 Centre for Pharmacy, University of Bergen, Bergen, Norway; 3 School of Pharmacy, Newcastle University, Newcastle Upon Tyne, UK; 4 School of Healthcare, University of Leicester, Leicester, UK

**Keywords:** Care homes, Medicines reconciliation, Residential homes, Transitionary care

## Abstract

**Background:**

Residents of care or nursing homes are at a higher risk of medication-related harm, especially during care transitions. No medicines-related intervention has been identified that supports the safe transition for these residents moving into their residence following hospital discharge. A model of care integrating a number of intervention components is suggested to be most effective

**Aim:**

To investigate, via a systematic review and meta-ethnography, the factors which influence the impact of medicines related interventions.

**Method:**

In December 2020, Pubmed, MEDLINE, EMBASE, PsycINFO, and CINAHL Complete were systematically searched. All studies reporting on medicines-related interventions for residents following hospital discharge were included. Quality assessment was undertaken with a validated tool. Meta-ethnography was used to investigate the factors which influenced how interventions did, or did not work. Findings were mapped to a validated conceptual framework for integrated care.

**Results:**

From 3884 studies, nine met the inclusion criteria and were analysed. These were generally of medium quality (n = 6). The interventions were diverse: some tackled the transition process, some tackled follow-up care and some interventions involved both. The meta-ethnography, using the a priori conceptual framework, captured factors reported within the studies that influenced implementation, delivery and/or outcomes.

**Conclusion:**

The review and synthesis informed the development of a conceptual model for transitionary care for this population group. Researchers and decision-makers can use this as a tool to understand their local context and inform future intervention design, implementation and evaluation in this clinical area.

**Supplementary Information:**

The online version contains supplementary material available at 10.1007/s11096-022-01507-3.

## Impact statements

- The systematic review and meta-ethnography aimed to identify the factors which influence how the delivery and engagement of medicines-related interventions at hospital to care home transitions.
- No individual medicines-related intervention has been identified that supports safe medicines use during transition for this population.
- Integrated care models with numerous intervention components are suggested to be most likely to achieve positive outcomes.
- Findings have informed a conceptual model for transitionary care for this population to support safe medicines use. The model can be used for future intervention design, implementation and evaluation.

## Introduction

Care or nursing home residents are a recognised vulnerable group due to their complex health and social care needs. Age, altered pharmacokinetics and pharmacodynamics, cognitive status, levels of comorbity and polypharmacy are just some factors which increase the risk of harm that can arise from medication use and medication-related processes [1–4]. A recent review found adverse drug events in care homes have been reported at 1.89 to 10.8 per 100 resident-months [5]. Medication safety in this context has been reported to be primarily influenced by five factors: persons, which includes the residents, nursing and clinical staff; organisation, such as the extent and effectiveness of interprofessional collaboration; tools and technology available and in use; tasks, for instance workload and time pressures; and environment, that acknowledges issues such as staff distraction [5].

Hospital admissions and transitions between care settings are also highly experienced by residents [1, 3]. It is well acknowledged that during hospital admissions and discharges, patients experience significant disruption in all aspects of their daily life [3, 6]. In particular, medicines-related problems are prevalent where discontinuity occurs in the communication and coordination of care between settings [7]. Medication errors for residents during transitions from one care setting to another have been reported to occur in 13–31% of transitions [8], with a quarter of these potentially leading to patient harm [9]. Spinewine et al*.* explicate that interventions to optimise medication use in care homes, not exclusively at care transitions, can be implemented at different levels of the healthcare system; the micro level, referring to the care home, their organisation and practitioners involved, and/or the meso and macro levels, which are strategies external to the care home but impact on their practice [2].

A systematic review about interventions to improve transitional care between hospitals and care homes with nursing found three studies focussed on medication lists, concluding that pharmacist-led review may reduce transition-related medication errors [1]. The authors were unable to identify any intervention that clearly improved the communication of accurate and appropriate medication information shared between care homes and hospitals.

A comprehensive, multifaceted concept of transitional care that broadly outlines the way that health care can be structured and delivered to reduce medication-related harm during transitions for this population is lacking. Valentijin et al*.* opines that integrated health systems have the potential to efficiently improve the access, quality and continuity of care being delivered, especially for people with complex needs [10]. Authors provide a conceptual framework to clarify the perceived ambiguous phenomenon of integrated care, which suggests that integration needs to be pursued at different levels (*micro* -individal practitioners, residents, patients, carers; *meso*—organisations such as the hospitals, care homes, general practitioner practices, and *macro*—systems made up of structures, processes and techniques within which the organisations work and interact) within a system to enable care continuity and coordination for individuals and populations [10]. A model of transitionary care that acknowledges the complexity of health care delivery and need for integration, would be valuable to outline best practices, key service or intervention principles and to identify how these can fit across the system, and across organisational and professional interfaces. The most recent Medical Research Council (MRC) guidance for the evaluation of complex interventions emphasises that interventions need to be viewed as ‘events in systems’, calling upon researchers to more thoroughly investigate contexts and consider the dynamic interaction of intervention components *in-situ* [11]. Authors describe context as ‘*any feature of the circumstances in which an intervention is conceived, developed, implemented and evaluated’* [11].

On this basis, this systematic review and meta-ethnography aims to identify these features or factors which influenced how the medicines-related interventions worked, or not to support the safe transitions of residents, once they are discharged from a hospital into their care homes. The focus was on whether the intervention was delivered and engaged with as intended to achieve the planned goals. Meta-ethnography is a seven-phase, theory-based, interpretive methodology for qualitative evidence synthesis. It involves systematic comparison of data from primary qualitative studies to identify and develop novel interpretations rather than aggregate findings [12]. In accordance to the revised MRC guidance [11], meta-ethnography was the selected synthesis to capture ‘clues’ (concepts, themes, metaphors) from included studies to better understand how and under what circumstances the interventions bring about change. We have for this purpose, considered that any information reported by authors, in their description of settings, intervention design, delivery, etc., as qualitative data. As such, this review is not limited to qualitative primary studies alone.

### Aim

To investigate, via a systematic review and meta-ethnography, the factors which influence the impact of medicines related interventions.

## Method

The meta-ethnographic systematic review is registered with the international prospective register of systematic reviews (PROSPERO register reference CRD42020221536) and has been conducted in accordance with the Preferred Reporting Items for Systematic reviews and Meta-Analyses (PRISMA) checklist [13] and the Meta-ethnography reporting guidance (eMERGe) [12].

### Search strategy

The electronic databases Pubmed, Ovid MEDLINE and EMBASE, PsycINFO, and CINAHL Complete (EBSCOhost) were comprehensively and systematically searched 2-15th December 2020 by one author [JK]. Bibliographies of all included studies were manually searched. No limit on the publication date was applied.

### Eligibility criteria

The ‘population, intervention, comparison, outcomes’ framework [14] was used to define the inclusion and exclusion criteria (Table 1).

**Table 1:** Details about the inclusion and exclusion criteria used to frame the search stratgey

Inclusion criteria	P: Study population includes patients over 65-years transferred into nursing/care/residential homes I: Any study including a medicines-related intervention provided by any healthcare professional during transition Studies where the interventions are conducted in nursing/care/residential homes settings C: This was not specified O: Studies reporting qualitative and quantitative data related to rehospitalisation, the economic, clinical and humanistic parameters Studies reporting implementation and outcomes related to design and delivery of the intervention All types of study design All articles written in English with no date restriction
Exclusion criteria	P: Study population is under 65-years I: Interventions delivered by non-healthcare professionals Interventions delivered outside the nursing/care/residential home setting O: Studies reporting data not related to implementation, process or final (economic, clinical, humanistic) outcomes Published in a non-English language, Unable to retrieve full text, abstracts, Study protocols, conference documents and ongoing research

The search strategy was developed in Pubmed with the aid of an expert librarian, with MeSH and search terms adapted accordingly for the different databases. Search terms were based on the following:

(Care or nursing or residential or skilled-nursing or assisted-living or Age)

AND (Facili* or Home or Long term or Old Age)

AND (reconciliation or Review or counselling or History)

AND (Admission or Admit or transfer or transition* or Discharge or entry or enter*)

AND (Drug* or Medicine* or Medication* or Pharmaceutical)

Search strings across each of the databases are included in the Supplementary material A.

### Study selection

All eligible studies were exported to EndNote X9 and duplicates removed. Two authors [JK, JB] independently reviewed the titles and abstracts. All studies reaching consensus and those not rejected with certainty, were retrieved for full independent review by two authors [JK, JB]. Where discrepancies occurred, a third author was consulted [DW].

The bibliographies of all final studies were manually searched to identify further studies.

### Data collection process

Two authors [HN, AR] read and re-read the studies independently to gain familiarity. Data extraction was undertaken employing a customised data extraction sheet. The extracted data from studies included: study and author details; details of the intervention delivered (where the intervention involved changing the transition process (between hospital and care home). There was further categorisation using the system reported and utlised by Hesselink et al*.* [15] that includes intervention elements relating to information, communication, and coordination of care; where the intervention included an additional follow-up component (based in the care home alone), the additional care was also categorised, (*e.g*. medicines reconciliation); outcomes reported (processual and primary and secondary measures); and verbatim textual data from the studies which provided information about the factors which influenced how an intervention was was delivered and engaged with as intended to achieve the planned goals. In many cases, this was within the narrative or discussion of the studies rather than formal outcome data. Authors [HN, AR] convened to discuss this stage and gain consensus, a third author was consulted where necessary [DW].

Given the research approach to this review and meta-ethography, where the emphasis is on capturing information about reported features or factors of the context impacting the intervention, no quality assessment of the studies was deemed necessary.

### Analysis and interpretive synthesis

The 7-phases of meta-ethnography by Noblit and Hare [16] were applied to this review:

1. Getting started, 2. Deciding what is relevant to the initial interest, 3. Reading the studies, 4. Determining how the studies are related, 5. Translating the studies into one another, 6. Synthesising translations, and 7. Expressing the synthesis.

The verbatim textual data from the nine primary studies was captured in the data extraction sheet and collated to make up the first-order constructs of this review. The data were compared to determine how they are related; in doing so, the concepts raised by each study’s original authors were reviewed to build deeper meanings around the research question (phase 4). It was during this phase, an a priori conceptual framework for integration [10] was applied to the extracted data. This involved iterative cycles of referring to the data in the extraction sheet, mapping it to the constructs of the framework and reading and re-reading the original studies to ensure the context was considered and checking for further data that fit, or did not fit with the framework. This ‘translation of the studies into one another’ (phase 5) was specifically conducted through the chronological consideration of each study and its data as it mapped to the integration framework; here, data from study 1 was compared with those of study 2 (and so forth). As each study was compared, similar data were grouped, reviewed and refined to produce the coherent and distinct overarching themes and subthemes (second-order constructs). These were then mapped into constructs of the integration framework, i.e., clinical integration, professional integration, etc. [10] (these were the a priori third-order constructs as per the meta-ethnographic approach). Two authors [HN, AR] independently undertook all stages of the meta-ethnographic synthesis and met to reach consensus, involving a third author [DW] where necessary.

There was a deviation from the specific recommendations for conducting meta-ethnographies [16] in that the first order constructs were not limited to verbatim quotes captured solely from qualitative research. Instead, the authors of this review were open to extract the verbatim deductions, opinions, narrative and discursive statements of the authors of the included studies. This strategy aimed to capture new and deeper insights on a complex problem that has yet to be fully answered in the current research. The subsequent synthesis is therefore a product of this sense-making exercise, and offers hypotheses about the phenomenon of interest to be later empirically investigated and tested.

## Results

**Fig. 1:**
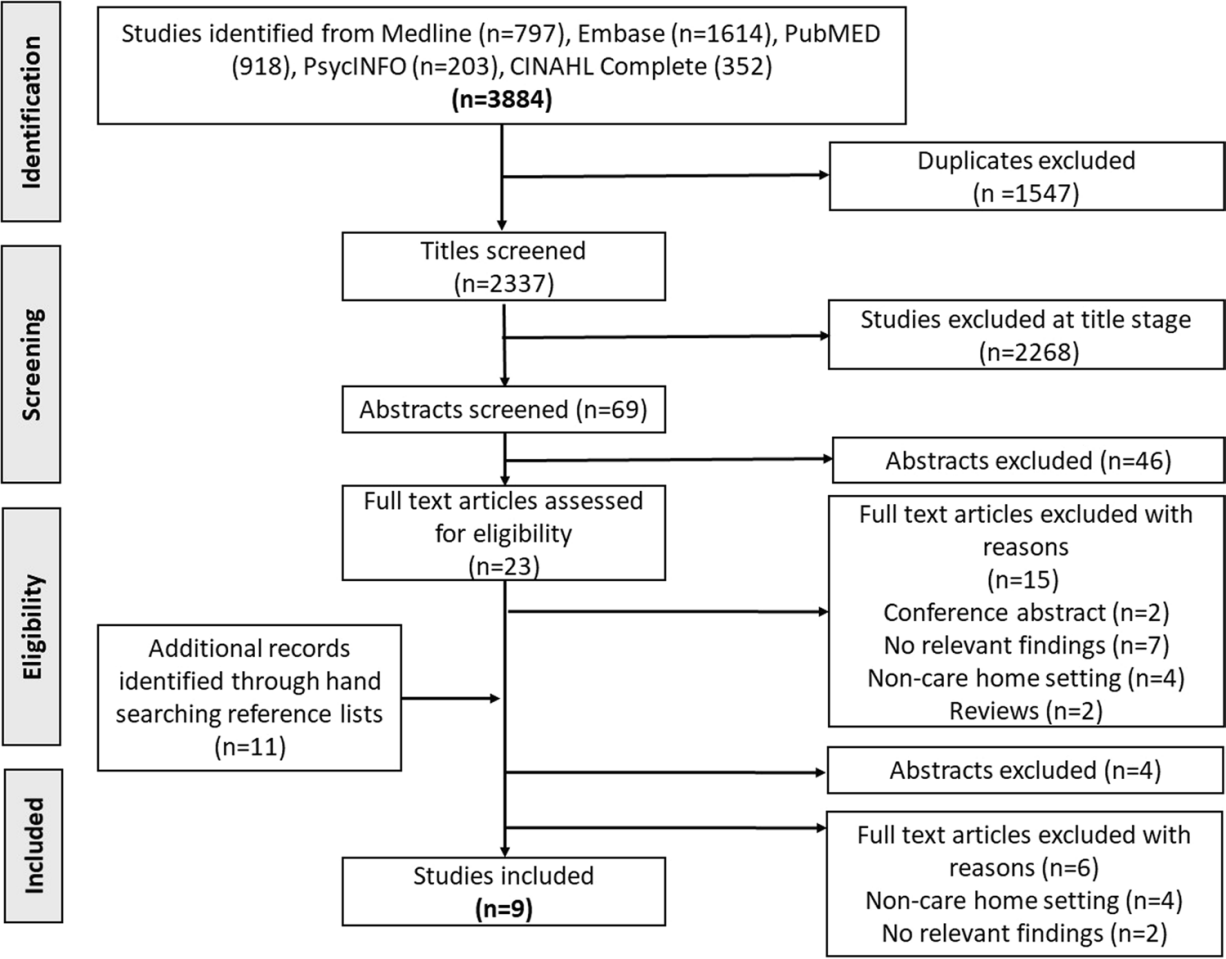
PRISMA flow diagram of included studies

A total of 3884 studies were obtained across the database searches. Figure 1 summarises the results from each stage of the study screening and selection process to yield a final nine studies meeting the inclusion criteria for analysis and synthesis.

### Study details and characteristics

There was a range of designs (Table 2) adopted across the studies, including: one RCT [17]; three pre-post- intervention evaluations [18, 20, 25]; two qualitative studies [19, 22]; two service evaluations reporting process outcomes [21, 23] and one quality improvement project [24]. There was some overlap in these categories, e.g. one of the pre-post- intervention evaluations was a quality improvement project [25]. The work had mainly been conducted in USA (n = 6) [18, 19, 21, 22, 24, 25], and one study in each of the following; Taiwan [20], Spain [23] and Australia [17].

**Table 2:** Information about study details, measures and outcomes

Study author, date	Setting	Study design	Intervention components	Measures	Outcomes
Crotty et al. [17]	Older adults making first transition into long-term residential care facility. 3 hospitals in South Australia	Randomised controlled trial	Transition process (information, communication and coordination of care) Follow-up care (medication review and case conference)	Quality of prescribing with the Medication Appropriateness Index (MAI) Emergency department visits, hospital readmissions, adverse drug events, falls, worsening mobility, worsening behaviours, increased confusion and worsening pain	No change in MAI in intervention group, but had worsened in the control group. Intervention group also experienced protective effect on worsening pain and hospital usage but did not differ on other outcomes
Boockvar et al. [18]	Nursing home and its primary referral hospital in New York City, USA	Pre-post intervention study	Follow-up care (medicines reconciliation)	Occurrence of discrepancy-related ADEs	Odds of having a discrepancy-related ADE were significantly lower in the post-intervention group compared with the pre-intervention group
Vogelsmeier et al. [19]	Eight Midwestern nursing homes, USA	Qualitative study	Follow-up care (medicines reconciliation)	Observations and interviews (n = 46) with nursing home staff	Nursing home staff undertook medicines reconciliation but staff varied on how they processed transferred information to identify errors. Perceptions between staff about medicines reconciliation also differed
Kuo et al. [20]	Nursing home affiliated with a hospital in Taiwan	Pre-post intervention study	Transition process (coordination of care) Follow-up care (medicines reconciliation)	Number and type of medication discrepancies at medicines reconciliation	There were less documented discrepancies in the study period. Eleven of the 12 (91.7%) harmful discrepancies in the study period were corrected in a timely manner
Krol et al. [21]	High-risk older adults, Hospital and skilled nursing facilities (SNF) in North Carolina, USA	Service evaluation and cohort study	Transition process (information, communication, coordination of care) Follow-up care (multi-component SNF evaluation including clinical handover and medicines reconciliation)	Recording of the types of nurse practitioner interventions All cause 14- and 30-day readmission rates	NPs conducted consultations with patients about rehabilitation, advanced care planning, contingency planning and made medication recommendations to the primary care team about pain, bowels and delirium, a further 44% (n = 134) recommendations were non-medication related Intervention patients had similar 14 but lower 30- day readmission rates than the control group
Patterson et al. [22]	2 residential care facilities and 2 skilled nursing facilities in the Midwest, USA	Qualitative study	Follow-up care (resolving medication discrepancies)	One focus group with registered nurses (n = 5), one community pharmacist, one admissions administrator and one general administrator	Nursing homes require accurate and timely information during transitions. There is incompatible formats of information between care settings. Nursing homes require a specific communication workflow between settings to resolve errors as effectively and efficiently as possible
Koprivnik et al. [23]	Five elderly nursing homes, Spain	Service evaluation	Follow-up care (medicines reconciliation)	Records from the centralised database were investigated to quantify and classify medication reconciliation errors detected during transitions of care Assess if error frequency was associated with polypharmacy or type of transition, and analyse type of medication involved	At least one medication error was found in 16% of 2123 care transitions. Wrong dosing (48%) and medication omissions (31%) were the most frequently detected errors. High-risk medication was involved in 40% of the cases. A positive association between polypharmacy and frequency of reconciliation errors was found. Different types of transitions did not show a difference in error frequency
Achilleos et al. [24]	12 acute care facilities and 7 nursing facilities in Virginia, USA	Quality improvement project	Transition process (communication, information, coordination of care)	Assessed time to undertake medication administration and time to order entry	After implementation, the mean time for order entry decreased 106%; on average orders were entered 6 min prior to patient’s arrival at the nursing facility. There was a decrease in average medication administration delay by 68% Medication discrepancies were identified in 51% of transferred patients including inappropriate doses, omissions, duplicate medications, unnecessary medications and a need for additional medications
Anderson et al. [25]	Skilled nursing facility in Alabama, USA	Pre-post implementation design/Quality improvement project	Follow-up care (medication reconciliation)	Assess all cause readmissions	An absolute hospital readmission rate decreased from 19.2% pre-implementation to 13.5% post-implementation. This was not significant, but achieved the quality measure of timely medications reconciliation at admission into the facility

### Interventions to address transitions of residents into care homes

The interventions were diverse in nature. Some addressed or modified elements of the transition process as categorised by Hesselink et al*.* [15] (i.e., relating to information, coordination of care, or communication) and introduced a follow-up intervention in the care home (n = 3) [17, 18, 21]. In two of these, all three elements of the transition process were considered, i.e. communication, information and coordination of care [17, 21], and the other amended the coordination of care of the transition process [20]. One study reported an intervention that tackled all three elements of the transition process with no additional follow-up component [24]. The remaining five studies reported a newly introduced follow-up intervention with no changes made to the transition process itself [18, 19, 22, 23, 25]. Of the studies where a follow-up intervention was introduced, medicines reconciliation was the specific intervention or formed part of a multi-component intervention.

The outcomes that were investigated and reported were similarly diverse; quantitative clinical [17, 21, 25], qualitative [19, 22], and processual data outcomes [17, 18, 20, 21, 23, 24]. However, the remit of this review was not to identify and critique the evidence for an intervention(s), thus the outcome data has not been interrogated to search for associations with patient impact. Instead, the outcome data has been used as a form of evidence in the following meta-ethnographic synthesis to understand the features or factors which impacted how the intervention was conceived, delivered, and engaged with in its particular context.

### Meta-ethnographic synthesis

Supplementary material B captures the key concepts (first-order constructs) from the studies that were considered during reciprocal translation and refutation towards the development of the themes (second-order constructs), and how they relate to the constructs of the framework for integration (third order constructs). The abridged version of these findings are presented in Table 3.

**Table 3:** The second order (themes) and third order (a priori concepts from the integration framework) constructs from the meta-ethnography

Third order integration concepts		Second order constructs or themes
Clinical integration	Functional	Appropriately trained and suitably accountable clinical staff undertaking roles, e.g. Medicines reconciliation
		Accurate and timely recording of clinical information
		Accessibility to complete and accurate patient/resident information
	Normative	Patient-provider relationship that strives to involve the patient with person-centred delivery
		Clinical prioritisation to acknowledge high-risk patients/residents for intervention targeting
Professional integration	Functional	Professional roles and responsibilities that are not reliant on individuals, but are integral to holistic healthcare package
		Clear professional role and responsibility descriptions that are mandated
	Normative	Interprofessional buy-in to coordinate services
		Personal relationships/understanding between professionals
		Established understanding of roles and responsibilities
Organisational integration	Functional	Integration of interventions within existing organisational pathways
		Establishing an organisational workflow to support timely communication and care coordination
		Mandated inter-organisation relationships between hospital and nursing homes
	Normative	Established informal relationship or affiliation between hospital and nursing home
		Embedding an inter-organisational culture of collaboration that is not reliant upon individuals
		Engagement activities and monitoring of performance within organisations
System integration	Functional	Digital integration where electronic health records are shared across care settings
		Access and reference to the same resources for patients across settings
		Electronic functionality to facilitate automation of interventions
		Real-time data transfer with read–write capability
		Supportive remuneration for collaborative working
		Well-resourced organisation to support service delivery
	Normative	Shared goals around patient safety and patient care

**Fig. 2:**
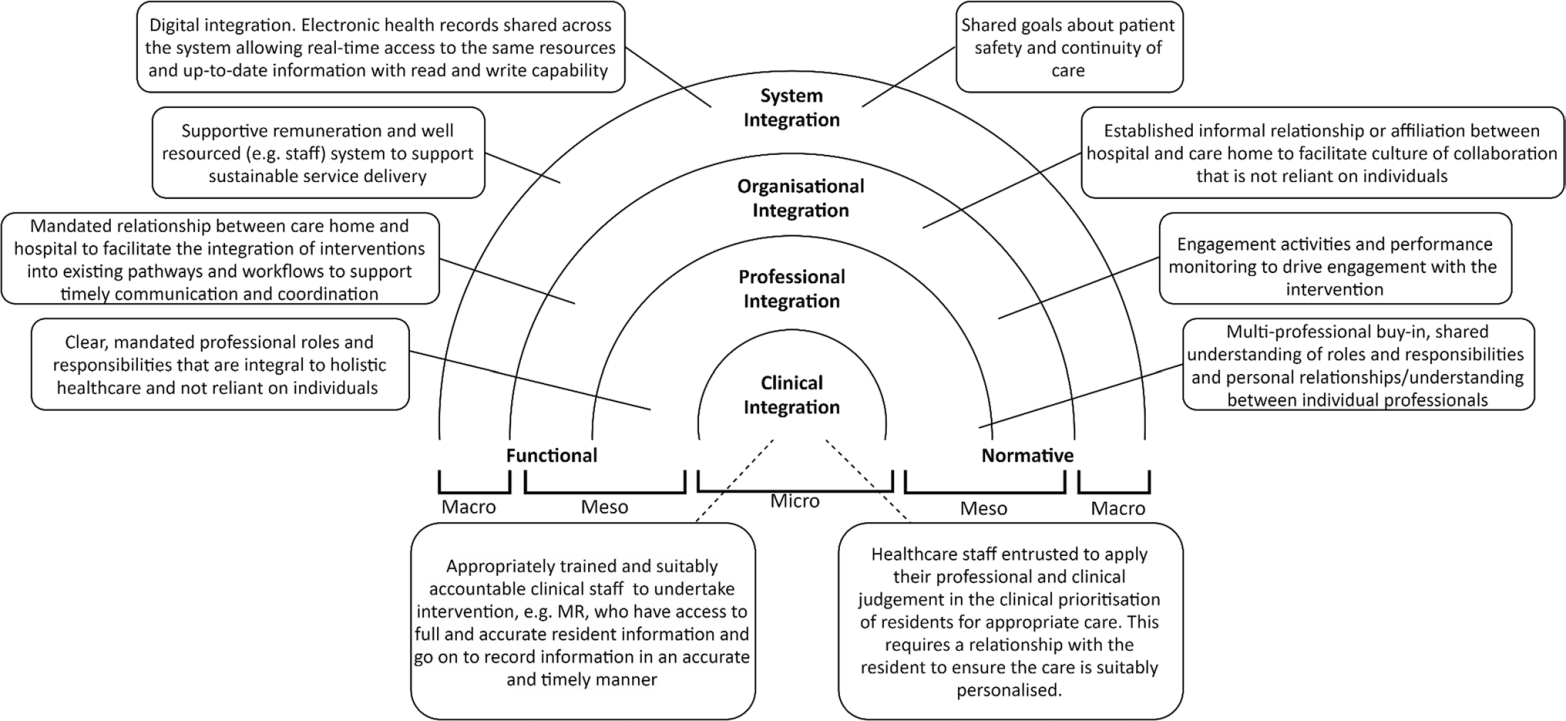
A conceptual framework for an integrated model of care to support safe transitions for residents as they move into care homes post hospital discharge (MR: Medicines reconciliation)

Figure 2 aims to illustrate the interconnection of the generated themes or hypotheses across a conceptual framework for the integrated model of care to support safe transitions of care for residents as they transfer into care homes following hospital discharge. The only data that did not fit in this framework was a subtheme pertaining to the need for the intervention to be informed by evidence, which is highlighted in Supplementary material B.

### Clinical integration

Many of the studies made reference to the required cognitive skills and recognised accountability of healthcare staff undertaking clinical interventions with residents during their transitions [17–21, 25]. Medicines reconciliation was the most frequently mentioned post-discharge follow-up intervention; in five of the studies, this was undertaken by a pharmacist [17, 18, 20, 23, 24], in another three studies it was nurses with the appropriate training and assigned responsibility [19, 21, 25], and in the remaining study it was unclear whether it was done by a pharmacist or nursing staff [22]. Where the skills or accountability of staff were deficient, it was reported that the integrity, and therefore the quality, of the clinical intervention was detrimentally impacted [19]. The need to have access to the most up-to-date and timely clinical information to inform care decisions was widely reported, with many of the intervention designers striving to achieve this for the intervention providers [17, 19, 20, 22–24]. Those providers of care were deemed to be more effective if they knew or had engaged previously with the resident, as they were able to be more person-centred [19]. There was also mention that those at the care interface with residents were well placed to execute their professional and clinical judgement, in order to prioritise clinical care appropriate to the residents’ needs and situation [17, 18, 20, 21].

### Professional integration

Across the studies there were diverse approaches to improve the holistic care being provided to residents as they transitioned from hospital, into their place of residence. Most reported the continuity of care as being dependent upon single individuals or professionals [17, 19, 21, 23–25], to the extent that in the absence of this individual (for annual or sick leave) the residents were deprived of the care or intervention offered to support their transition [21, 23, 25]. In some studies, additional clinical staff formed part of the intervention, for instance a ‘transition coordinator’ [17, 24]. In one study, there was a call for clear role descriptors for the nurse practitioners in skilled nursing facilities to clarify and recognise the care that is provided to residents by these specific clinical staff [25]. The general focus of this was placed on the professionals and their roles and responsibilities, indicating that clarity and descriptors could be a necessity to ensure residents do not experience variable and discontinuous care. It was acknowledged in some of the studies that interprofessional commitment and appreciation of others’ roles and responsibilities were crucial to addressing the issues during transitions [17, 19, 21, 24]. One study postulated that personal-professional relationships have the capacity to improve continuity of care for residents, as clinical staff feel more empowered and confident in managing handover of care from individuals they are professionally acquainted with [19].

### Organisational integration

Some of the studies reported making use of existing clinical staff, resource and infrastructure, whereas others described standalone interventions. The most isolated, extra-organisational intervention required additional ad hoc funding and staffing [21]. Using existing clinical staff and infrastructure meant that staff involved in the delivery of an intervention were well acquainted with the contexts, professionals and processes of primary and secondary care settings. This facilitated the clinical care provision for residents that was the most person-centred [19, 22–24]. Similarly, some studies described the testing of new workflows for the intervention, or making use of existing workflows. New workflows required additional activities such as training, engagement and raising awareness [17, 21]. Existing workflows also posed their own challenges, where deficiencies in organisational process, resource or infrastructure contaminated the intervention being tested; for instance, poor timeliness of information transmission during transitions or poor medicines reconciliation practices, leading to inaccurate information being transmitted [17, 22, 24]. In either case, established organisation workflows to address issues in the timeliness and accurateness of information and coordination of care is warranted. One study stated that the partnering of hospitals with skilled nursing facilities would facilitate the integrity of workflows to reduce hospital readmissions [25]. Whereas, other studies made reference to less formal affiliations, rather than partnerships, to improve the continuity of care [20, 21, 23]. Two studies described the interprofessional collaboration occurring across primary and secondary settings to support intervention provision for residents [21, 25]. Lastly, the study that tested the intervention that was most separated from existing practice and workflows, also described engagement activities and longitudinal monitoring of performance in the attempt to continually assess and optimise delivery and impact [21].

### System integration

In the attempt to address issues around timeliness and accurateness of information transfer, studies cited system-based solutions featuring digital integration and access to real-time data with read–write capability [17, 21–24]. One study also described the benefits of having access to the same medicines formulary, or even the ability to view formularies across settings, to help with the coordination of care [22]. Some of the studies reported how elements of usual care, or the intervention, were not provided due to aforementioned problems with workflows and availability of resources and staffing. It was suggested that automation of some of these elements (for instance, the automatic transmission of full, up-to-date discharge information) would improve the continuity of care on a system-wide level [17, 18, 21].

Many of the studies reported a lack of appropriate clinical staffing to support safe transitions of residents from hospital to their place of residence [19, 21, 24, 25]. This problem is worsened by the aforementioned dependence on individual professional roles and responsibilities, where absence of staff leads to sporadic care provision. The suggestion is that the system needs to be well resourced and staffed, to support coherent care provision, and this should be supported with conducive remuneration packages or structures [17, 21].

Lastly, shared goals within the healthcare system around care provision was reported to underpin positive engagement and participation in transitional care interventions [21, 25].

## Discussion

The meta-ethnographic approach has provided insight into elements of a system or context which warrant consideration when developing services to improve the transition of care for care home residents as they are discharged from hospitals. Findings have been inductively generated from existing studies and deductively organised using a validated integration framework [10]. The product is a set of interlinked concepts or hypotheses at various levels of a system illustrating the need to consider an intervention within the dynamic environment it will be delivered.

In comparing these findings with other work, many studies outside of the specific review criteria exist and report on the individual insights packaged into our conceptual framework. For instance, Elliott et al*.* described that reliance on locum doctors to manage medication information following hospital discharge may be detrimental to patient safety as they would be unfamiliar with the resident and therefore potentially less person-centred [26]. Similarly, a videoconferencing intervention between hospital-based teams with clinicians in post-acute care sites was reported to facilitate personal professional relationships and a sense of teamwork to support interprofessional care provision [27].

However, there is other research that offers concepts that could add granularity to this framework. For instance, the systematic review by Murray et al*.* considers the level of care involvement for older people during transitions [3]. Patient safety is recognised to be enhanced where there is involvement and engagement of the person in their care. However, the ability of older people in this vulnerable group to participate in such a way, poses a challenge. The lack of the involvement of patients or care home residents in our included studies is potentially a manifestation of this challenge. Murray et al*.* recommend that intervention designers should endeavour to recognise and address those factors influencing potential involvement to ensure care is as personalised and safe as possible [3].Unfortunately, our findings have not provided any evidence or information on how best to do this, so more work in this area is warranted.

These examples demonstrate that the framework has the possibility to be enriched through current empirical studies to provide a more comprehensive theoretical perspective of the phenomenon of interest. However, we would suggest that in its current form, the framework is more valuable to support onward intervention design and evaluation, forming the first steps (i.e., 1. identifying the evidence base and, 2. identifying and developing appropriate theory) in the recommended guidance for the design and delivery of complex interventions [28]. Next steps could be to scrutinise the pertinent barriers and facilitators to addressing the problem of interest within a specific context, e.g. local healthcare system. An exercise could be undertaken to understand the most important contextual factors that would most favourably support an effective intervention.

A recent systematic review about successful transitions for older people, although not specifically living in care homes, found that interventions bridging the transition including self-management, medicines reconciliation and telephone follow-up were most effective to support older patients’ medication continuity [29]. Authors were unable to identify how to best engage patients and stakeholders across the system to facilitate implementation and delivery of these intervention components [29]. The findings of our review provides some factors or features of a system that could be considered to facilitate improved intervention delivery and engagement.

Our work is strengthened by the systematic approach to finding literature and considering studies as a whole in our synthesis to answer our research question. However, our framework is theoretical, based on the evidence derived from included studies and produced from the interpretive meta-ethnographic approach. As a research team, the general premise and motivations of our work is to understand how to provide better health and social care for patients. We have an ingrained perspective that an integrated approach will best achieve this, which has influenced our inductive approach in using a conceptual framework for integration to frame our findings. This may have limited our analysis and interpretation, since we may have been more inclined to support our biases rather than be open to those which challenge our preconceptions. We concede that further in-depth scrutiny of the evidence, alongside engagement and consultation with stakeholders, could help address our own biases but also help identify causal mechanisms contributing to outcomes as afforded by a realist approach. This could have produced more insightful, fuller theories that could be tested in future work.

## Conclusion

The findings from this systematic review and meta-ethnography provide researchers and decision-makers with a conceptual model of transitionary care to support safe transitions for residents as they move back into care homes, following hospital discharge. The model presents some features or factors across the system that have been identified to impact how interventions are delivered and engaged with. Future intervention designers and commissioners are recommended to consider the wider context within which an intervention of this type will be delivered towards addressing the specific local challenges and exploit the extant facilitators towards achieving more successful outcomes.

## Supplementary Information

Below is the link to the electronic supplementary material.

Supplementary file1 (DOCX 14 KB)

Supplementary file2 (DOCX 22 KB)
