# Supplementary material for: Contextual factors influencing medicines-related interventions to support safe transitions for care home residents post hospital discharge: a systematic review and meta-ethnographic synthesis
Source: Int J Clin Pharm. 2022 Nov 17;45(1):26–37. doi: 10.1007/s11096-022-01507-3 (PMC9938806; doi:10.1007/s11096-022-01507-3)
Supplement: Supplementary file 1 — Supplementary file1 (DOCX 14 KB) [file 11096_2022_1507_MOESM1_ESM.docx]

Search strings across the databases

| **Database** | **PubMed** | **Ovid EMBASE** | **Ovid MEDLINE** | **PsycINFO** | **CINAHL complete (EBSCOhost)** |
| --- | --- | --- | --- | --- | --- |
| **Search terms** | (Care[Title] OR ((((Care[Title] OR nursing[Title] OR residential[Title] OR skilled-nursing[Title] OR assisted-living[Title] OR Age[Title]) AND (Facili*[Title] OR Home*[Title] OR Long term[Title] OR Old Age[Title])) AND (reconciliation OR Review OR counselling OR History)) AND (Admission OR Admit OR transfer OR transition* OR Discharge OR entry OR enter*)) AND (Drug* OR Medicine* OR Medication* OR Pharmaceutical) | ((Care or nursing or residential or skilled-nursing or assisted-living or Age) and (Facili$ or Home$ or Long term or Old Age)).ti. and (reconciliation or Review or counselling or History).af. and (Admission or Admit or transfer or transition$ or Discharge or entry or enter$).af. and (Drug$ or Medicine$ or Medication$ or Pharmaceutical).af. | ((Care or nursing or residential or skilled-nursing or assisted-living or Age) and (Facili$ or Home$ or Long term or Old Age)).ti. and (reconciliation or Review or counselling or History).af. and (Admission or Admit or transfer or transition$ or Discharge or entry or enter$).af. and (Drug$ or Medicine$ or Medication$ or Pharmaceutical).af. | TI ( Care OR nursing OR residential OR skilled-nursing OR assisted-living OR Age ) AND TI ( Facili* OR Home* OR Long term OR Old Age ) AND ( reconciliation OR Review OR counselling OR History ) AND ( Admission OR Admit OR transfer OR transition* OR Discharge OR entry OR enter* ) AND ( Drug* OR Medicine* OR Medication* OR Pharmaceutical ) | TI ( Care OR nursing OR residential OR skilled-nursing OR assisted-living OR Age ) AND TI ( Facili* OR Home* OR Long term OR Old Age ) AND ( reconciliation OR Review OR counselling OR History ) AND ( Admission OR Admit OR transfer OR transition* OR Discharge OR entry OR enter* ) AND ( Drug* OR Medicine* OR Medication* OR Pharmaceutical ) |
| **Hits** | 918 | 1614 | 797 | 203 | 352 |
