# Supplementary material for: Contextual factors influencing medicines-related interventions to support safe transitions for care home residents post hospital discharge: a systematic review and meta-ethnographic synthesis
Source: Int J Clin Pharm. 2022 Nov 17;45(1):26–37. doi: 10.1007/s11096-022-01507-3 (PMC9938806; doi:10.1007/s11096-022-01507-3)
Supplement: Supplementary file 2 — Supplementary file2 (DOCX 22 KB) [file 11096_2022_1507_MOESM2_ESM.docx]

The key concepts (first order constructs) supporting the evidence-based intervention subtheme which does not fit within the conceptual framework for integrated care

| **Subtheme** | **Key concepts from studies** |
| --- | --- |
| Evidence-based intervention | The transition pharmacist coordinated an evidence-based medication review.[14]  The institute for Healthcare Improvement’s State Action on Avoidable rehospitalisations program provided a framework for addressing the hospital to Skilled Nursing Facility transitions of care.[18]  Larrabee’s six-step model for evidence-based practice change was used as the conceptual framework to guide the quality initiative.[22] |

*The key concepts (first order constructs), themes (second order constructs) and a priori concepts for integrated care (third order constructs)*

| **Third order integration concepts** | | **Second order constructs or themes** | **First order constructs or key concepts** |
| --- | --- | --- | --- |
| **Clinical integration** | **Functional** | Appropriately trained and suitably accountable clinical staff undertaking roles, e.g. Medicines reconciliation | Use of a pharmacist with specialised understanding of medication use in older adults appears to hold great promise.[13]  Pharmacist-conducted medication reconciliation in combination with physician communication can improve medication safety and patient outcomes at the time of transfer between facilities.[14]  Six nursing homes (75%) assigned medicines reconciliation to any nursing staff admitting the resident (RN or LPN), whereas only two nursing homes (25%) assigned medicines reconciliation exclusively to RNs.[15]  Many RNs spoke about the value of detailed transfer documentation to help understand the resident’s clinical condition. The RNs often reviewed transfer information in detail to identify medication order inconsistencies they perceived to ‘happen all the time’.[15]  A few were hesitant to question the intent of transfer orders. In one instance, an RN new to her nursing home role commented, ‘I don’t know this hospitalist very well, so I’m guessing he ordered what he wanted.’[15]  When performing medication reconciliation, most would focus on discharge documents to note any inconsistencies. The remaining transfer documents were often not reviewed.[15]  Many nursing home safety practices are more than simple tasks; instead these practices are clinical processes that require complex cognitive skills to carry them out. Safety practices such as medicines reconciliation require nursing staff who possess the necessary cognitive skills to ensure medication order discrepancies are appropriately identified and managed.[15]  To perform medicines reconciliation, the pharmacist compared the patient’s medications prior to admission with the current new prescriptions.[16]  The results of this study provide evidence that a pharmacist-implemented medication-reconciliation program can prevent potential adverse events to nursing home residents during transfer.[16]  NP completed inpatient consultation consisting of a thorough medication review, a geriatric assessment, and recommendations to the inpatient primary team.[17]  By having pharmacy personnel take ownership of medication order entry prior to patient arrival, nurses were able to redirect their focus to direct patient care.[20]  She (the NP) was versed in the multiple regulations of the SNF environment due to her past experience. Prior to implementation of the project, she was guided on the purpose of the project, the medication reconciliation flowsheet, the evidence researched to formulate the flowsheet, and the work flow process to be followed with each patient admission during the project time frame.[21] |
|  |  | Accurate and timely recording of clinical information | There were discrepancies between the medications sent with the patient and the medications listed on the discharge summary in 57.1% (intervention) and 48.1%(control) of patients.[13]  16.1%(intervention) and 24.1%(control) of patients were discharged without a physician’s discharge summary to accompany nursing summary.[13]  Discrepancies between hospitalists and primary care physicians’ medication orders cause delays because nursing home staff must follow-up and clarify the medication orders between physicians in different settings.[18]  It took 4 hours after SNF admission for hospital discharge orders to be entered into the SNF EMR.[20] |
|  |  | Accessibility to complete and accurate patient/resident information | Many RNs spoke about the imperative to know the resident’s clinical history as a way to make sense of their medication orders.[15]  The reasons for physician acceptance of the pharmacists’ suggestions were usually related to the physician not knowing the details of the patients’ previous medication use.[16]  Outdated information from a hospital or inconsistent medication history challenge nursing home staff.[18]  Primary care physicians may have not received discharge information, and their knowledge of patient’s medical history could be critical to resolve discrepancies.[18]  Standardised hospital transfer sheets increase the likely hood of nursing homes receiving complete information at intake, reducing the need for follow-up phone calls that further delay the medicines reconciliation process.[18]  At first admission to the NH, the pharmacist used the electronic health record (EHR), electronic prescription application (EPA), internal geriatric health record (GHR) and did a ‘Brown bag’ review.[19]  Another commonly observed type of delay occurred because hard copies of prescriptions for controlled substances were not sent with the patient during transfer from the acute care facility, and the pharmacy could not fill the order without a hard prescription due to regulatory constraints.[20] |
|  | **Normative** | Patient-provider relationship that strives to involve the patient with person-centred delivery / involvement | Caregiver gaining consent.[13]  LPNs often spoke about having to make decisions about which discrepancies to communicate. ‘Sometimes the hospital changes their meds and the nurse has to decide if those changes should be changed back…we know them [residents] better and sometimes know how they handle [respond to] certain medications’.[15]  NP educated patients and caregivers using a pamphlet on expectations for short-stay SNF placement and answered questions on the rehabilitation process.[17]  Based on the initial assessment, the NP addressed rehabilitation potential, contingency plans if the rehabilitation goals were unmet, and advanced care planning.[17] |
|  |  | Clinical prioritisation to acknowledge high-risk patients/residents for intervention targeting | Target patients receiving multiple medications or medications whose interruption is likely to be associated with immediate withdrawal and/or relapse symptoms (e.g. opioids, antidepressants).[14]  First-time admission to a long-term care facility is a key transition in which fragmentation of care may occur and communication between providers is often suboptimal.[13]  Polypharmacy is usually common in the elderly, which comprise the largest population in long-term care units, so performing medication reconciliation in such units may be one way of reducing medication errors.[16]  Based on the higher-risk for readmissions for patients discharged to SNF.[17]  The ideal HOPE patient is at high risk for readmission and for complications during the hospital to SNF transition. The HOPE used a consultation model, whereby patient selection was at the discretion of the primary medical team. this methodology promoted clinical judgement in identifying the appropriate patients.[17]  We are piloting a second NP consultation visit for patients who appear to be at risk for readmission during the initial SNF follow-up.[17] |
| **Professional integration** | **Functional** | Professional roles and responsibilities that are not reliant on individuals, but are integral to holistic healthcare package | Pharmacist transition coordinator.[13]  On transfer to residential care 62.5% patients in intervention group and 76% in the control group were allocated to a new family physician, as they had moved homes.[13]  A multidisciplinary staff developed a medication reconciliation protocol. First nursing home residents returning from a hospital stay were identified within 1 day of their return. Second, a pharmacist conducted a reconciliation in which drugs ordered when the resident returned to the nursing home were compared with those received before the hospitalisation, and any discrepancies were identified and recorded. Each discrepancy was categorised as an omission, addition, dose change, or substitution. Third, prescribing discrepancies were communicated to the nursing home physician via a communication form. Finally, the physician reviewed the form, recorded whether he or she intended to take any action, and signed it. The form became part of the nursing home pharmacy record.[14]  LPNs more often focused on medication orders they perceived as either inappropriate or necessary for the nursing home. These decisions were seemingly influenced by organisational rules and regulations. RNs more often focused on medication orders they perceived to be high risk or those not making clinical sense.[15]  Physicians providing care in the SNF were not associated with the discharging hospital healthcare system.[17]  Post discharge evaluation focused on communicating critical hospital care information directly to the clinical staff, identifying medication errors in the transition process, and confirming SNF awareness of outpatient follow-up appointments.[17]  NP communicated with a variety of SNF providers, rehabilitation therapists, social workers, and transportation coordinators. The NP reconciled high-risk medications, discussed rehabilitation progress with therapy staff, and updated SNF clinicians on hospital course, pending laboratory tests, and goals of care.[17]  Reasons for lack of SNF follow-up included clinical absence of NP.[17]  The pharmacist isn’t present all weekdays and therefore depends greatly on the collaboration with other NH staff.[19]  The SNF pharmacist was chosen as the lead for the pilot project due to that individual’s unique position of being able to access the EMRs of both acute care facility and the SNF and accessibility to both care teams.[20]  To implement this program across all facilities within the health system, a dedicated pharmacist would be needed.[20]  During the project, a 4-day period, where the NP was off for personal time and no medication reconciliation or NP stabilisation visit was performed, 6 patients were admitted to the SNF and three of those were returned to the hospital within a 30-day period.[21] |
|  |  | Clear professional role and responsibility descriptions that are mandated | Federal health policy advocacy to reduce restrictions on what NPs may perform in the SNF setting.[21] |
|  | **Normative** | Interprofessional buy-in to coordinate services | Transferring information on medications to care providers in the long-term care facility, including the nursing staff, the family physician, and the accredited community pharmacist.[13]  Transition pharmacist coordinated case conference involving him/herself, the family physician, the community pharmacist, and a registered nurse at the facility within 14-28 days of the transfer.[13]  In the hospital, all study participants had received care form an experienced specialist medical team that included geriatricians, pharmacists, nurses, and allied health care workers, as reflected in the low levels of inappropriate prescribing on discharge.[13]  RNs understood the realities of limited physician and pharmacy resources in the nursing home and fragmented medical care between settings.[15]  A multidisciplinary team with a focus on medication management during the transition from acute care to SNF was formed.[20]  Additional key stakeholders from both facilities were engaged in designing a new, efficient process.[20] |
|  |  | Personal relationships/understanding between professionals | ‘I don’t know this hospitalist very well, so I’m guessing he ordered what he wanted.’[said by a RN] [15] |
|  |  | Established understanding of roles and responsibilities | Differences emerged between RNs and LPNs when performing medication reconciliation. Differences were related to their perceptions about medication reconciliation, as well as their actions when performing the process. The RNs’ and LNs’ perceptual differences were related to why medication reconciliation was being performed, including their role in the process and the kinds of medication order discrepancies they identified.[15]  RNs talked about resident safety and their responsibility to pull the ‘big picture’ together because physicians and pharmacists were often not onsite at the nursing home when the transfers occurred.[15]  Majority of LPN staff viewed medicines reconciliation as a task, citing it as ‘something I have to do’.[15]  RN and LPN staff differ in both what they communicate and to whom, and provides insight into what nursing home nurses communicate to physicians that may in turn influence what physicians prescribe.[15]  Understanding distinct differences between RN and LPN roles is important to assuring the right clinical staff are appropriately assigned to the right clinical process.[15]  Hospital-based physicians had limited understanding of SNF capabilities and workflow, hindering optimal transitional care and patient preparedness for SNF rehabilitation.[17] |
| **Organisational integration** | **Functional** | Integration of interventions within existing organisational pathways | It may be possible to train pharmacy technicians or involve existing nursing or medical staff in documenting reconciliation.[14]  Good primary care and care coordination should include regularly performed and documented medication reconciliation.[14]  LPNs are expected to function in highly clinical roles. As such, perhaps consideration should be given to enhanced education and training for LPNs to build the necessary cognitive skills to work in the complex nursing environment.[15]  Components of HOPE will be incorporated into the workflow of hospital staff…to further disseminate the HOPE care transition process throughout the hospital while limiting the NP involvement to higher risk patients.[17]  The integration of our hospital pharmacists in the NH teams, having full access to both the general health systems and the NH’s electronic medical charts, enables the former to act as a liaison between different sites of care.[19] |
|  |  | Establishing an organisational workflow to support timely communication and care coordination | Transition pharmacist coordinated an evidence-based medication review that was performed by the community pharmacist contracted to the facility within 10-14 days of transfer.[13]  ~72hrs after discharge the NP provided a SNF evaluation and staff communication by phone or in-person. This timing was found to be preferred over earlier follow-up because SNF nursing, therapy and medical personnel had an opportunity to evaluate the patient and identify questions or issues.[17]  The HOPE also added the consultation order to the admission pathway for patients with hip and femur fractures.[17]  Optimal time for the inpatient HOPE consultation requires consideration. Timing the initial HOPE inpatient evaluation to occur just prior to discharge proved difficult to coordinate, as it required the NP to constantly monitor changing discharge plans. Also the consults deviated from typical follow-up timeline (i.e. within 24hours), leading to the clinicians feeling that the consult services were not rendered in a timely manner.[17]  Nursing home staff have established policies and communication protocols to be followed to resolve medication discrepancies as a process to obtain signature approvals. Participants described some practical challenges such as staff working different shift schedules between settings and timing of a new admission that make timely communication more difficult because others may not be immediately available.[18]  Participants described problems when physicians do not follow the required procedures in the nursing home setting. If a physician provides an order out of sequence, the nursing home must ask the physician to complete the order again.[18]  During the pharmacy processing of the medication order, if a discrepancy was identified there were multiple steps for clarification…this often caused a significant delay, as nursing staff had to handle medication pass, provide direct resident care, and receive clarification from inpatient and/or SNF providers prior to order entry into the EMR.[20]  A goal of this process was to develop an efficient workflow between nursing staff, pharmacy staff, and providers to reduce the time delays identified in the previous process.[20]  In this new process, the pharmacist had direct contact with inpatient and SNF providers for clarifications, eliminating the multiple steps nursing personnel previously had to complete.[20] |
|  |  | Mandated inter-organisation relationships between hospital and nursing homes | To comply with federal mandates, SNFs must partner with referring hospitals to reduce hospital readmissions.[21] |
|  | **Normative** | Established relationship between hospital and nursing home | Nursing home was affiliated with the hospital.[16]  Joint SNF-hospital interprofessional workgroup convened to improve the hospital to SNF transition.[17]  When a patient is admitted into one of these NHs, all aspects related to their pharmaceutical care become the responsibility of the hospital pharmacist.[19]  We designed a transitional care model that combined both prehospital discharge and postdischarge components aimed at improving quality of the transition and minimising hospital readmissions, without relying on an established network of SNF partners.[17] |
|  |  | Embedding an inter-organisational culture of collaboration that is not reliant upon individuals | A multidisciplinary team moved the new program forward and provided quarterly reports to hospital leadership about the outcomes. The team consisted of an NP, a geriatrician, hospitalists, geriatrics fellows, nursing leadership representatives, and an administrative leader.[17]  The medical director for the facility made rounds at the SNF one partial day per week along with two part-time NPs who could not provide a consistent schedule due to working in an office practice.[21] |
|  |  | Engagement activities and monitoring of performance within organisations | The HOPE group met weekly to develop strategies for improving patient identification, program utilisation, and outcome monitoring.[17]  The HOPE program required a consultation order from the primary hospital physician; consultations were promoted through key stakeholder engagement and frequent outreach to hospitalists, house staff, case managers and therapists.[17]  Several advanced degree candidates (nursing and hospital administration) contributed; examples include creating the educational pamphlet for patients and families about SNF rehabilitation and developing a business plan.[17] |
| **System integration** | **Functional** | Digital integration where electronic health records are shared across care settings | Faxed a medication summary.[13]  Improved information exchange between facilities requires health information exchanges and electronic health record systems.[18]  Future policies need to focus on decreasing the digital divide between nursing homes and hospitals in order to improve the accuracy of exchanged information.[18]  The SNF pharmacist was chosen as the lead for the pilot project due to that individual’s unique position of being able to access the EMRs of both acute care facility and the SNF and accessibility to both care teams.[20]  The ability to access both inpatient and SNF medication records allowed the pharmacist to collaborate with both inpatient and SNF physicians to determine appropriate medication regimens.[20] |
|  |  | Access and reference to the same resources for patients across settings | Confusion with prescriptions can occur because hospitalists use a different institutional formulary that does not match a resident’s Medicare Part D or other insurance formulary.[18] |
|  |  | Electronic functionality to facilitate automation of interventions | All patients were transferred with a nursing summary; however, 9 (16.1%) and 13 (24.1%) of patients in the respective groups were discharged without a physician’s discharge summary to accompany the nursing summary.[13]  Efforts to engineer electronic medical records should incorporate medicines reconciliation procedures so that reconciliation can be carried out in an automated fashion.[14]  We are currently evaluating a program that electronically alerts physicians of patients with high readmission risk scores and suggested interventions, including a HOPE consultation.[17] |
|  |  | Real-time data transfer with read-write capability | Substantive real-time communication between hospital and SNF physicians was not occurring.[17]  NP documented the post hospital discharge SNF communication in the health system’s electronic health record, making it accessible to inpatient and outpatient care providers.[17]  Participants described discrepancies related to last minute changes that the hospitals do not include on the transfer sheet. Many times hospital providers do no explain last minute changes that may not be consistent with paperwork they sent to nursing homes a few days prior.[18]  The active medication list was updated in the patients’ EHR and EPA.[19]  Our NH pharmacists always kept an updated medication list in the EHR where it could be consulted by anyone working in our public health system.[19] |
|  |  | Supportive remuneration for collaborative working | Community pharmacists are paid to perform an annual medication review for residents of long-term care facilities.[13]  Pharmacists are reimbursed for medication reviews on a bed-per-year basis rather than on the basis of per patient service, which may have limited community pharmacists’ provision of medication services and attendance at case conferences.[13]  Reimbursement considerations required consult authorisation from a physician or an advanced practice provider, limiting the ability to use therapists or case managers for patient selection.[17]  Identifying a viable funding model for the program was challenging. For sustainability the HOPE required financial investment from the hospital. The hospital provided support through an innovation grant mechanism, which allowed for data collection and case reviews that informed the development of the program.[17]  The recruitment of the NP, with the ability to bill professionally to offset salary expenses, was an important factor in sustaining the program, but the important work of follow-up after discharge to SNF has not been billable according to Medicare rules.[17] |
|  |  | Well-resourced organisation to support service delivery | RNs understood the realities of limited physician and pharmacy resources in the nursing home.[15]  RN staffing continues to decline in nursing homes.[15]  Annual costs of the program include 1.0FTE for an NP, 0.1FTE for an administrative manager, and 0.1FTE for a medical director.[17]  This process (entering admission data) is owned by nursing personnel who had multiple other duties, with an average of 15 patients per nurse.[20]  Lack of medical providers and processes to ensure adequate medication reconciliation and resultant medication changes at the point of admission are a serious concern.[21]  The facility added a dedicated full-time NP in late 2016.[21] |
|  | **Normative** | Shared goals around patient safety and patient care | Challenges arose in educating the primary team on appropriate referral criteria and maintaining engagement with HOPE.[17]  We also engaged the therapists, nurses, and case managers to identify high-readmission-risk patients and advise the clinical team on the benefits of a HOPE consultation.[17]  To comply with federal mandates, SNFs must partner with referring hospitals to reduce hospital readmissions.[21] |
